# Evaluation of two local cowpea species for nutrient, antinutrient, and phytochemical compositions and organoleptic attributes of their wheat-based cookies

**DOI:** 10.3402/fnr.v60.29600

**Published:** 2016-01-20

**Authors:** Rufina N. B. Ayogu, Ngozi M. Nnam, Mirabel Mbah

**Affiliations:** Department of Home Science, Nutrition and Dietetics, University of Nigeria, Nsukka, Nigeria

**Keywords:** childhood and adolescent malnutrition, cowpea, micronutrients, *oraludi*, *apama*

## Abstract

**Background:**

Childhood and adolescent malnutrition is a function of inadequate intake. Cookies are favourite snacks of children and adolescents.

**Objective:**

This work determined the nutrient, antinutrient, and phytochemical compositions of two local cowpea (*oraludi* and *apama*) flours and evaluated the organoleptic properties of their wheat-based cookies.

**Design:**

The two local cowpea species were processed into flours separately and blended with wheat on a 56-g protein basis. Chemical compositions of the processed cowpea flours were analysed using standard methods. Organoleptic attributes were evaluated with a nine-point Hedonic scale. Statistical analysis, which involved mean and standard deviations, were computed by analysis of variance, and Duncan's new multiple range tests were used to separate and compare group means of sensory evaluation data, with significance accepted at *P*<0.05.

**Results:**

The results revealed that *oraludi* had superior percentage values compared to *apama* in protein (26.22 and 20.88), fat (7.98 and 6.65), and ash (3.81 and 3.13), while *apama* proved superior in moisture (9.76 and 7.82), crude fibre (5.49 and 4.91), and carbohydrate (54.09 and 49.26). The values were higher for *oraludi* than *apama* in iron (8.62 and 6.49 mg), zinc (1.61 and 0.95 mg), and beta-carotene (223.24 and 190.63 mg) but lower in sodium (34.79 and 56.72 mg), potassium (25.73 and 30.65 mg), phosphorus (13.35 and 18.26 mg), thiamine (5.33 and 9.41 mg), vitamin C (16.63 and 21.09 mg), and vitamin E (0.51 and 0.67 mg). *Apama* had 0.06 mg phytate, 0.09 mg oxalate, 15.22 mg tannins, 3.59 mg flavonoids, and 0.19 mg saponin. *Oraludi* had 0.03 mg phytate, 0.32 mg oxalate, 15.94 mg tannins, 3.14 mg flavonoid, and 0.13 mg saponin. Mean scores of general acceptability for wheat:*apama* (80:20) and wheat:*oraludi*:*apama* (60:20:20) cookies (7.71 and 7.41) were superior (*P*<0.05) to others.

**Conclusions:**

*Oraludi* and *apama* proved nutrient dense. Their use improved the acceptability of some of the wheat-based cookies. Use of these local cowpeas in cookie production is, therefore, encouraged.

Childhood and adolescent malnutrition is increasing especially among the low- and middle-income countries of the world. Most of the affected children suffer from hidden hunger because their diets are mainly carbohydrate based. Protein and micronutrient deficiencies are often major nutritional problems of these children. This is a result of growth spurt and inadequate nutrient intake. Ochola and Masibo (1) asserted that in developing countries, the diets of school-age children and adolescents are very limited in diversity. The pattern is characterised by minimal intake of animal foods, fruits, and vegetables and high consumption of calorie-rich processed foods.

Snack consumption has been increasing as a result of urbanisation and increase in the number of working mothers (2). Snacks are small, light, very handy, and simple meals which do not replace main meals. They add to the total food and nutrient intake of individuals. Most of them are baked, and this enhances their keeping quality (shelf life). Most snacks especially those commercially prepared are made from wheat alone. Cookies are one of the most popular baked wheat products, widely consumed due to its ready-to-eat nature, low cost, and longer shelf life (3).

Consumption of cereal foods, such as biscuits and bread, is very popular in Nigeria especially among children and adolescents. Children and adolescents are the main consumers. In support of this, Olapade and Adeyemo (4) affirmed that cookies, otherwise known as biscuits, are popular cereal foods, commonly consumed by the populace, especially the preschool and school-aged children in Nigeria. In Nigeria, cookie consumption is continually growing, and there has been increasing reliance on imported wheat for the production of biscuits and baked products (5). Most developing countries are interested in the possibility of replacing the wheat needed for baking foods, wholly or partly, with flour obtained from home-grown products (6).

Complementation of cereal-based foods with protein sources such as legumes has received considerable attention (4, 7–9). Legumes are important sources of low-cost vegetable proteins and micronutrients when compared to animal products such as meat, fish, and egg. The expensive nature of these animal foods makes them less preferred to plant sources.

Indigenous legumes, therefore, are important sources of affordable alternative protein to poor people in many resource-poor countries where they are predominantly consumed.

In developing countries, research attention is being paid to better utilisation of legumes in addressing protein malnutrition and food security issues. This is because legume protein is high in lysine, which is deficient in most cereals. Ihekoronye and Ngoddy (10) observed that plant proteins have limited amino acids; cereal flours are poor in lysine but rich in sulphur containing amino acids (methionine and cystine). The addition of legumes is an important means of improving the nutritional quality of cereal foods. It was observed that not only are legumes excellent sources of essential minerals but they are also rich in dietary fibre and phytochemicals, which may affect health positively (9).

It was observed that most school children (adolescents inclusive) who skipped breakfast consumed cookies as alternative. In some schools, the most frequent snack given to children is cookies. Some mothers use cookies to pacify their crying children.

To prevent malnutrition resulting from over consumption and over dependence on most wheat-based cookies, there is a need for protein complementation. Legumes have nutrients (protein and micronutrients) which can be used as complements for cereals such as rice, wheat, sorghum, and maize. The complementation of cereal foods with legumes results in the development of a new pattern of amino acid, such that the limiting amino acids are compensated for.

It is in light of this that this study was carried out to produce cookies capable of contributing reasonably to the daily recommended nutrient intake of children and adolescents; a strategy which ensures that protein and micronutrient deficiencies among this group is prevented and controlled. The use of local cowpea was to ensure diversity, accessibility, availability, and affordability.

## Materials and methods

### Source of materials

**Fig. 1:**
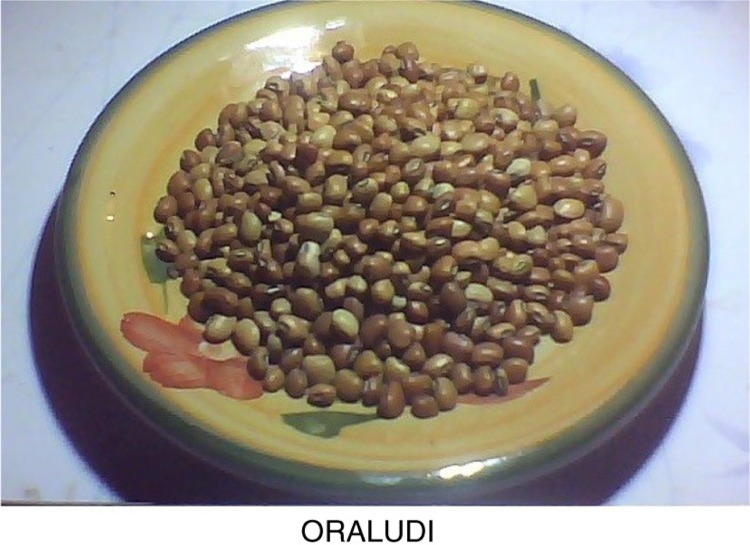
Sample of *oraludi* (*Vigna biflorus*) seeds.

**Fig. 2:**
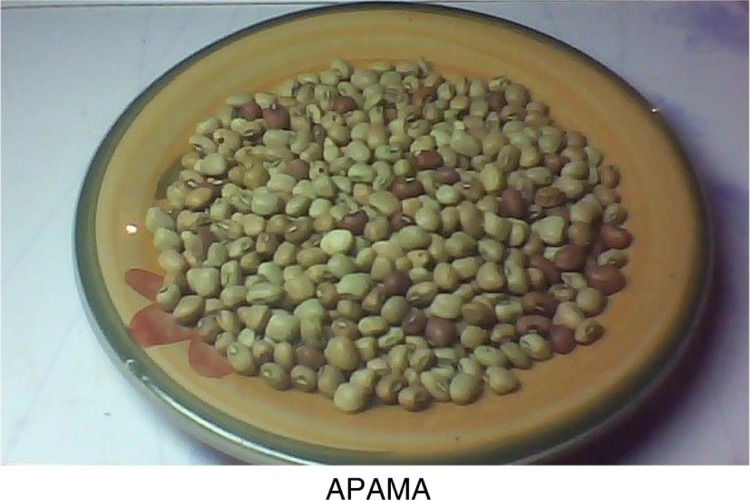
Sample of *apama* (*Vigna sinenssis*) seeds.

Wheat flour, local species of cowpea (*Vigna biflorus and Vigna sinensis*; local names: *oraludi* and *apama*), baking fat (margarine), granulated sugar, milk powder, egg, baking powder, and salt used for this work were purchased from *Ogige* market in Nsukka, Enugu State, Nigeria. Samples of *oraludi* and *apama* seeds are shown in Figs. 1 and 2.

### Preparation of the sample

**Fig. 3:**
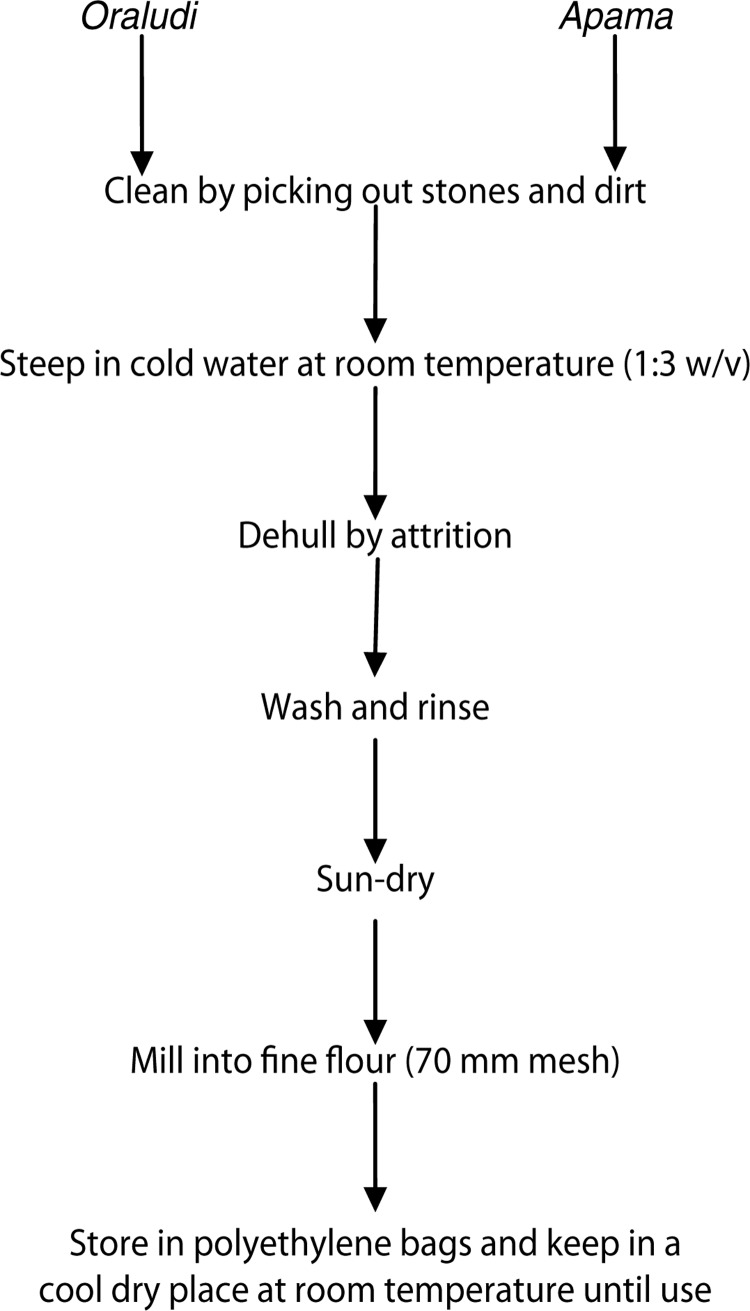
Flow chart for processing *oraludi* and *apama* flours.

Five kilograms of *oraludi* and *apama* seeds each were picked clean. Each portion was steeped in water at room temperature in the ratio of 1:3 w/v. The seeds were dehulled independently by attrition; washed, rinsed, sun-dried, and hammer milled with Thomas-Wiley Mill, Model ED-5, England, into fine flour (70 mm mesh screen). Twenty grams of the samples each was taken for chemical analysis. The remaining portions were packed separately in polyethylene bags and stored at room temperature in a cool dry place until required (Fig. 3).

### Formulation of composite flours

The crude protein of *oraludi* and *apama* flours was determined by micro-Kjeldahl method (11). The composites were formulated on 56-g protein basis in the ratio of 80:20, 70:30, 60:40, 80:10:10, 70:15:15, and 60:20:20 of wheat, *oraludi*, and *apama*. The protein quantity of 56 g, chosen as a basis for the formulation, was to provide the recommended protein to an adolescent male aged 16–18, and this will incidentally furnish the protein requirement of males and females of all age groups below this. This quantity is not too much for age groups below 16–18 because plant proteins have low bioavailability.

In all, nine composite flours were formulated. Table 1 shows the blends, ratios, and quantity of flours (grams) required to furnish 56 g of protein. Taking into account that 26.2 g of protein was obtained from 100 g of *oraludi* and 10.4 g from 100 g of wheat, then 80% of 56 g from wheat will be obtained from 430.8 g and 20% of *oraludi* will be obtained from 42.7 g as shown in Table 1.

**Table 1:** Blends, ratios, and quantity of wheat, *oraludi*, and *apama* flours

Composite blends	Ratio	Quantity (g)
Wheat (control)	100:00:00	538.5:0.0:0.0
Wheat+*oraludi*	80:20:00	430.8:42.7:0.0
	70:30:00	377.0:64.1:0.0
	60:40:00	323.1:85.4:0.0
Wheat+*apama*	80:20:00	430.8:53.6:0.0
	70:30:00	377.0:80.5:0.0
	60:40:00	323.1:107.3:0.0
Wheat+*oraludi*+*apama*	80:10:10	430.8:21.4:26.8
	70:15:15	377.0:32.1:40.3
	60:20:20	323.1:42.7:53.7

The portions of the respective blends in grams were determined by simple proportion. The amount of protein to be supplied by each composite was determined from the ratio of the blends (using 56 g protein as 100%). The amount required to furnish each requirement was based on the protein composition of the composites. Half of this quantity was used in the production of the cookies.

### Chemical analysis

The methods described by Association of Official Analytical Chemists (11) were used for triplicate chemical analyses for proximate mineral and vitamin compositions. These have been described elsewhere (12–16). Caloric value was calculated by Atwater factors (a gram of protein, carbohydrate, and fat yields 4, 4, and 9 kcal, respectively). Phytate (17), tannins (18), oxalate (19), flavonoid (20), and saponin (21) were determined by the respective methods. Phytate zinc molar ratio (PZMR) (an index of zinc absorption) was calculated using the following International Zinc Nutrition Consultative Group (22) formula:

[Phytic acid (mg/100 g)÷molecular weight of phytic acid (660)]÷[Zinc (mg/100 g)÷molecular weight of zinc (65.4)]

A PZMR of less than five depicts a relatively high absorption of 55% from the diet in which zinc is contained. A ratio of 5–15 indicates moderate absorption of 35%, and a ratio of more than 15 shows low absorption of 15% (23).

### Production of cookies

A modification of the recipe described by Oyewole et al. (24) was used in the production of the wheat-based cookies. Margarine and sugar were mixed together in a Kenwood mixer at a moderate speed until a light and fluffy cream was obtained. Edible portion of a medium-sized egg and milk powder were added while mixing and this was allowed to mix very well. The composite flour, baking powder, and salt were added slowly into the mixture to complete the dough formation. Some flour was sprinkled on a flat board and the dough rolled on it with a wooden rolling pin to form a uniform thickness of 0.4 cm. Circular cookies of 5.52 cm in diameter were cut and placed on greased baking pans and baked in a gas oven at 150°C for 15 min. The ingredients and the respective quantities used in the production of the cookies are presented in Table 2.

**Table 2:** Quantity of ingredients used in the preparation of cookies

Ingredients	Control (100%wheat)	Wheat+*oraludi* A	Wheat+*oraludi* B	Wheat+*oraludi* C	Wheat+*apama* A	Wheat+*apama* B	Wheat+*apama* C	Wheat+*oraludi*+*apama* A	Wheat+*oraludi*+*apama* B	Wheat+*oraludi*+*apama* C
Wheat flour (g)	269.3	215.4	188.5	161.6	215.4	188.5	161.6	215.4	188.5	161.6
*Oraludi* flour (g)	0.0	21.4	32.1	42.7	0.0	0.0	0.0	10.7	16.1	21.4
*Apama* flour (g)	0.0	0.0	0.0	0.0	26.8	40.3	53.7	13.4	20.2	26.9
Sugar (g)	60.0	60.0	60.0	60.0	60.0	60.0	60.0	60.0	60.0	60.0
Beaten whole egg (g)	60.0	60.0	60.0	60.0	60.0	60.0	60.0	60.0	60.0	60.0
Baking powder (g)	10.0	10.0	10.0	10.0	10.0	10.0	10.0	10.0	10.0	10.0
Salt (g)	2.5	2.5	2.5	2.5	2.5	2.5	2.5	2.5	2.5	2.5
Milk Powder (g)	100.0	100.0	100.0	100.0	100.0	100.0	100.0	100.0	100.0	100.0
Margarine (g)	80.0	80.0	80.0	80.0	80.0	80.0	80.0	80.0	80.0	80.0
Total dough weight (g)	536.8	504.3	488.1	471.8	509.7	496.3	482.8	507.0	492.3	477.4

For wheat+*oraludi* and wheat+*apama, A*=80:20, *B*=70:30, *C*=60:40. For wheat+*oraludi+apama, A*=80:10:10, *B*=70:15:15, *C*=60:20:20.

### Sensory evaluation

Thirty students were randomly selected by balloting from third- and final-year students of the Department of Home Science, Nutrition and Dietetics, University of Nigeria, Nsukka. The selection was based on their previous participation in similar works. Flavour, colour, taste, texture, and general acceptability of the cookies were evaluated by a nine-point Hedonic scale (25). The degree to which the products were liked was expressed as: like extremely (nine points), like very much (eight points), like moderately (seven points), like slightly (six points), neither like nor dislike (five points), dislike slightly (four points), dislike moderately (three points), dislike very much (two points), dislike extremely (one point). Like extremely to like slightly were considered as good while dislike slightly to dislike extremely were taken as poor. Neither like nor dislike showed that the quality of the product was neither good nor bad and was regarded as neutral.

Sensory assessment was carried out in the Food Research Laboratory of the Department of Home Science, Nutrition and Dietetics, University of Nigeria, Nsukka. Adequate lighting and ventilation were ensured in the food laboratory. Movements and other sources of distraction were controlled. The judges were arranged in such a way that they were unable to see the grading of each other. The samples were presented in coded plain colour, flat plates. Each judge was provided with water to wash hands and a clean hand towel to dry them. A glass of water to rinse his/her mouth after each testing was also provided. The exercise was carried out between 12 noon and 2 p.m.

### Statistical analysis

Results were analysed with computer software: Statistical Package for Social Sciences (SPSS), version 17. Data were presented as mean±standard deviation. Analysis of variance was used for data relating to sensory evaluation and Duncan's new multiple range tests were used to separate and compare the group means. Significance was accepted at *P*<0.05.

## Results

Table 3 shows the proximate composition and caloric value of the two bean flours. The moisture content of *apama* flour was slightly higher (9.76%) than that of *oraludi* (7.82%). Protein of *oraludi* (26.22%) was superior to that of *apama* (20.88%). Fat content of *oraludi* was 7.98%, and *apama* had 6.65%. Ash content of *oraludi* and *apama* flours was 3.81% and 3.13%, respectively. *Apama* had higher crude fibre (5.49%) and carbohydrate content (54.09%) than *oraludi* (4.91 and 49.26%) but lower caloric value (359.4 Kcal) than *oraludi* (373.7 Kcal).

**Table 3:** Proximate compositions and caloric values of *oraludi* and *apama* flours (100 g)

Variables	*Oraludi*	*Apama*
Moisture (%)	7.82±0.14	9.76±0.35
Protein (%)	26.22±0.23	20.88±0.49
Fat (%)	7.98±0.41	6.65±0.29
Ash (%)	3.81±0.08	3.13±0.13
Crude fibre (%)	4.91±0.08	5.49±0.2
Carbohydrate (%)	49.26±0.57	54.09±0.67
Caloric value (kcal)	373.7 (1588.2 kJ)	359.4 (1527.5 kJ)

Values are mean±SD of triplicate determinations, 1 kcal equals 4.286 kJ.

The mineral and vitamin compositions of *oraludi* and *apama* flours (mg/100 g) were presented in Table 4. *Oraludi* flour had a higher iron content of 8.62 mg compared to *apama* (6.49 mg). It also proved superior in zinc (1.61 mg); *apama* had 0.95 mg. Sodium in *oraludi* was 34.79 mg, and in *apama*, it was 56.72 mg. There was a wide range of variation in their potassium and phosphorus compositions. Potassium and phosphorus in *apama* were 30.65 and 18.26 mg, respectively while *oraludi* contained 25.73 mg of potassium and 13.35 mg of phosphorus. Thiamine in *apama* (9.41 mg) was higher than that of *oraludi* (5.33 mg). Both varied in their beta carotene and vitamin C contents. *Apama* contained 190.63 mg (37.2 RE) of beta carotene and 21.09 mg of vitamin C. *Oraludi* had 16.63 mg of vitamin C and 223.24 mg (31.8 RE) of beta carotene. The two samples had varying amounts of vitamin E: *apama* had 0.67 mg, while *oraludi* had 0.51 mg.

**Table 4:** Mineral and vitamin compositions of *oraludi* and *apama* flours (100 g)

Variables	*Oraludi*	*Apama*
Minerals		
Iron (mg)	8.62±0.40	6.49±0.34
Zinc (mg)	1.61±0.08	0.95±0.06
Sodium (mg)	34.79±1.85	56.72±0.25
Potassium (mg)	25.73±0.38	30.65±1.40
Phosphorus (mg)	13.35±1.07	18.26±0.36
Vitamins		
Thiamine (mg)	5.33±0.24	9.41±0.11
Beta-carotene (mg)	223.24±2.32 (31.8 RE)	190.63±4.09 (37.2 RE)
Vitamin C (mg)	16.63±0.29	21.09±0.29
Vitamin E (mg)	0.51±0.08	0.67±0.00

Values are mean±SD of triplicate determinations.

Table 5 explains the antinutrients, phytochemicals, and PZMRs of the two local cowpea flours. *Oraludi* was composed of 0.03, 0.32, and 15.94 mg of phytate, oxalate, and tannins, respectively. *Apama* contained phytate (0.06 mg), oxalate (0.09 mg), and tannins (15.22 mg).

**Table 5:** Antinutrients, phytochemicals, and PZMRs of *oraludi* and *apama* flours (100 g)

Variables	*Oraludi*	*Apama*
Antinutrients		
Phytate (mg)	0.03±0.014	0.06±0.017
Oxalate (mg)	0.32±0.001	0.09±0.001
Tannins (mg)	15.94±0.079	15.22±1.99
Phytochemicals		
Saponin (mg)	0.13±0.022	0.19±0.017
Flavonoid (mg)	3.14±0.014	3.59±0.002
PZMR	0.002	0.006

PZMR=phytate zinc molar ratio. Values are mean±SD of triplicate determinations.

*Apama* proved superior in saponin (0.19 mg) and flavonoid (3.59 mg). *Oraludi* had 0.13 mg saponin and 3.14 mg flavonoid. PMZRs of *apama* and *oraludi* were 0.006 and 0.002, respectively.

Organoleptic attributes of the cookies were revealed in Table 6. Texture score for the cookies ranged from 4.21 in WA3 to 8.29 in WO3. Scores for colour ranged from 3.07 in WA1 to 7.07 in WA3. WA2 and WOA1 had comparable (*P*>0.05) mean colour scores of 6.40 and 6.43, respectively. Taste scores differed among the samples. WOA3 and WO3 had the highest (7.57) and lowest (3.50) scores, respectively, and these were significantly different (*P*<0.05). The taste scores of WA1 were similar (*P*>0.05) to WO1 and WOA3, but the flavour of the cookies varied. WA1 recorded higher score of 6.64 than others. The least score of 3.64 was found in WO3. General acceptability scores ranged from 2.71 in WO3 to 7.71 in WA1. General acceptability of WA1 (7.71), WOA3 (7.41), and WA2 (6.93) were similar (*P*>0.05) but significantly (*P*<0.05) higher than the control (6.36), WA3 (5.50), WO1 (6.48), and WOA2 (5.50). WO3 (2.71), WOA1 (3.93), and WO2 (4.36) were similar (*P*>0.05) but significantly (*P*<0.05) lower than others.

**Table 6:** Organoleptic attributes of the cookies

Blends	Texture	Colour	Taste	Flavour	General acceptability
W	4.57±2.03^a^	3.93±1.82^a^	6.50±1.09^b^	5.50±1.83^c^	6.36±1.65^b^
WA1	5.29±2.23^a^	3.07±2.51^a^	7.50±2.03^c^	6.64±2.5^c^	7.71±2.94^c^
WA2	6.79±1.92^b^	6.40±1.65^c^	5.04±1.69^b^	4.79±1.76^b^	6.93±2.53^c^
WA3	4.21±1.92^a^	7.07±2.02^c^	5.64±1.72^b^	5.29±1.82^b^	5.50±1.99^b^
WO1	5.93±1.9^a^	6.00±1.71^b^	7.47±0.94^c^	6.07±1.94^c^	6.48±1.87^b^
WO2	5.57±2.03^a^	5.03±1.82^b^	6.50±1.09^b^	5.00±1.83^b^	4.36±1.87^a^
WO3	8.29±2.03^b^	4.07±2.56^a^	3.50±2.53^a^	3.64±2.45^a^	2.71±1.97^a^
WOA1	5.79±1.93^a^	6.43±1.55^c^	4.44±1.69^a^	4.79±1.76^b^	3.93±2.51^a^
WOA2	7.22±2.19^b^	6.07±2.02^b^	5.64±1.82^b^	5.29±1.82^b^	5.50±1.99^b^
WOA3	5.00±0.9^a^	4.00±1.79^a^	7.57±0.94^c^	6.07±1.94^c^	7.41±1.87^c^

Values are mean±SD (*N*=30). Values with different superscripts in the same column are significantly different (*P*<0.05).

W=Wheat alone (control).

WA1=Wheat flour supplemented with *apama* flour in the ratio of 80:20.

WA2=Wheat flour supplemented with *apama* flour in the ratio of 70:30.

WA3=Wheat flour supplemented with *apama* flour in the ratio of 60:40.

WO1=Wheat flour supplemented with *oraludi* flour in the ratio of 80:20.

WO2=Wheat flour supplemented with *oraludi* flour in the ratio of 70:30.

WO3=Wheat flour supplemented with *oraludi* flour in the ratio of 60:40.

WOA1=Wheat flour supplemented with *oraludi* and *apama* flours in the ratio of 80:10:10.

WOA2=Wheat flour supplemented with *oraludi* and *apama* flours in the ratio of 70:15:15.

WOA3=Wheat flour supplemented with *oraludi* and *apama* flours in the ratio of 60:20:20.

## Discussion

Legumes are rich sources of protein and can reliably be employed to complement the protein of cereals. Noor Aziah (2) asserted that legumes can be used to provide amino acids such as lysine. Cereal-legume foods are known to be more nutrient rich than either of the two as a result of which it has received much attention recently (7, 26, 27).

The proximate compositions of the local cowpeas varied. Proteins of *oraludi* and *apama* (Table 1) were higher than the values reported for mungbean (16.10%), chickpea (19.90%) flour (2) and pigeon pea flour (8). Legumes are rich sources of protein, and even though they are second-class proteins, their availability and affordability are advantages over animal proteins. Animal proteins are often expensive and unaffordable by most families in low- and medium-income countries. Legumes, therefore, stand in the gap to ensure that protein is not omitted entirely in meals consumed in these families. Moreover, bioavailability of legume protein and most minerals can be improved by processing. Madukwe et al. (12) reported higher protein content in *oraludi* with fermentation. This was supported by Olapade et al. (28) and shows that products made from these legumes would contribute reasonably to the RNI of most age groups. Wheat flour has lower protein value of 10.4% (29).

Complementation of wheat flour with either of these two legumes or both is a means of not only increasing its protein value but also its micronutrient worth because most protein foods are carriers of micronutrients such as zinc, iron, and copper. Children and adolescents are particularly in need of protein and micronutrients. Most of them are micronutrient deficient due to multiple factors. Consumption of these products would therefore contribute immensely to the fight against these deficiencies. Higher protein content of legumes has nutritional significance. Moderate intake resulting from the consumption of snacks made from their blends with wheat will greatly increase the total dietary protein, micronutrient, and phytochemical intake of children and adolescents. In addition, its utilisation as a protein supplement in the production of wheat-based cookies will reduce the overdependence on the common wheat flour in use. Interestingly, we found some of these cookies were more generally accepted than the control which was made with wheat alone.

Ash content of both flours agreed with the report of Okpala and Okoli (8). Rich ash content of legumes has implication for mineral values. The fibre content of both flours was slightly higher than the previously reported contents of some legumes (2, 8, 12). These observed differences were attributed to differences in species, location, soil, as well as inter-laboratory differences in chemicals and methods. Fibre has been known for its health implications. It enhances intestinal motility and the activities of probiotics, reduces blood sugar and prevents colon and rectal cancers (30–32). The health of the children will therefore be protected and enhanced.

Fat is a good baking aid. The fat content of the samples has an advantage in baking. Fat is the main source of fatty acids which have been associated with wound healing (33) and immunity (34) and are very useful in the human body. These functions are crucial in the health of children because they are vulnerable to infections and injuries. Its high energy value is also an added advantage in curbing low weight-for-age among children and adolescents who have been shown to have high levels of physical activities. The fat level of the flours varied slightly from the findings of Vadivel and Janardhanan (30, 35) on velvet bean (6.3–7.4%).

The carbohydrate composition of the samples was found to be lower when compared to previous reports (8) on pigeon pea (69.43%) but similar to carbohydrate of certain underutilised food legumes (49.9–61.2%) (30, 35). Carbohydrates provide readily available glucose for energy production to meet the high activity level of children and adolescents. As most of them go to school and most times without breakfast, cookies made from this combination would be of immense help in furnishing these children with glucose and other nutrients to enhance brain work and sustenance for academic activities.

The vitamin C content of the legumes was high and unexpected. In support of this, Okwu and Orji (36) reported vitamin C content of 31.68 and 55.44 mg in processed *Vigna unguiculata* (ife brown and iron beans, respectively). Dietitians of Canada (37) also reported that Canadian Nutrient File (CNF) had the vitamin C value of cooked snow peas as 41 mg in half a cup of 125 ml capacity. In a comparative study on the vitamin C content of the food legume seeds by Moriyama and Oba (38), it was reported that total vitamin C and L-ascorbic acid in mung beans, green peas, broad beans, black soybeans, and adzuki beans remained even after boiling, suggesting that it is possible to obtain vitamin C from the cooked forms of these legume seeds. The high vitamin C reported in this study is a guarantee of better iron absorption from the legume products. Micronutrient content of the two local cowpea flours is an indication of good nutritional quality. Micronutrients play significant roles in numerous body processes. Consumption of foods rich in micronutrients and phytochemicals has been associated with good health. Iron, vitamin A, vitamin E, and zinc are known for their anti-anaemic, antioxidant, and immune booster effects. Sodium and phosphorous enhance fluid balance and nerve impulse transmission. Potassium ensures muscle cell contractility. Deficiency of phosphorus results in decreased growth, poor tooth development, and rickets (31–34, 39–42). Cookies made from these flours can furnish the body with significant amounts of these nutrients that can contribute effectively to meeting the daily recommended intake of the various nutrients.

Children need the protection that phytochemicals give. The consumption of legume-enriched cookies would make some contributions to health promotion and the protection they require against chronic non-communicable diseases as they advance in age. Flavonoids have the ability to stimulate human protective enzyme systems (35, 43). It has been affirmed that flavonoids have been associated with protection against bacterial and viral infections as well as cardiovascular diseases and cancers (36, 44–47). It, therefore, implies that consumption of these cookies will aid in the protection against infections which children are often exposed to. Matsuura (37, 48) reported that saponins exhibit a positive cardiovascular effect by lowering serum cholesterol.

The low PZMR of the flours depicts a good zinc absorption rate of 40–55% from the cookies and, therefore, ensures protection against diarrhoeal disease, a major health problem of childhood.

The various differences observed among the organoleptic attributes of the cookies were functions of individual differences in perception. Wheat complemented with *apama* (80:20), wheat complemented with *oraludi* and *apama* (60:20:20), and wheat and *apama* blend (70:30) had the highest but similar (*P*>0.05) general acceptability scores (7.71, 7.41, and 6.93, respectively). These had better (*P*<0.05) acceptability than wheat cookies (control). Wheat *oraludi* blend (80:20), wheat *apama* blend (60:40), and wheat *oraludi apama* blend of 70:15:15 were comparable (*P*>0.05) with wheat cookies in general acceptability. These cookies are good and would satisfy both the nutritional and organoleptic needs of the target consumers (children and adolescents). This is because they would make reasonable contributions to the food intake and, therefore, the nutrient pool of the children.

## Conclusions

This study showed that *oraludi* and *apama* are good sources of protein, micronutrients, carbohydrate, and phytochemicals with good organoleptic properties of their wheat-based cookies. Fortification of wheat flour with these legume flours in the production of cookies is a major step towards alleviating protein energy and micronutrient malnutrition because most children and adolescents depend on cookies as snacks.

## Recommendation

Production of these legumes on a large scale is encouraged. Individuals and industries are also encouraged to use the 80:20 wheat *apama*, 60:20:20 wheat, *oraudi* and *apama* and 70:30 wheat *apama* blends for cookie production as they will be accepted readily by the intended consumers.
